# Punishment by the Cangue in China

**Published:** 1881-10

**Authors:** 


### PUNISHMENT BY THE CANGUE IN CHINA.

This cangue is the main prop of the Mongolian order—the stocks, pillory and penitential cell of Kathay. It is merely a cage of crossbars, which are sometimes of iron, sometimes of heavy timber. The prisoner's body is inclosed in this cage, which reaches from his knees to his neck; his head and limbs are alone free, his hands being strapped to a bar. Now it is manifest that a criminal thus accoutered must be a prop and support of his own portable jail. A captive of Atlas, he carries about his own dungeon, and he cannot lie down to rest, but must pass whole days and nights on his feet, the poles attached to the cangue preventing him from lying down, while to the framework is fixed a placard inscribed with the wretch's name, offense and sentence. A cangue may weigh 100 pounds or only twenty, but in any case it is a dreadful punishment, kept on as it is for periods varying from six hours to six weeks. Imagine days and nights of cramps and sleeplessness, the harrassing stings of mosquitoes and other tormenting insects worrying the naked skin, and no hand to brush them away; the scorching sun, and no screen; the chilly night, and no covering; weariness, dizzy brains, limbs racked for dire fatigue, fever, delirium, the pressure of the hard yoke on the galled shoulders, the strangling collar, the agony of long want of sleep, the thirst, the shame! Men often go mad in the cangue, it is said; they fall asleep on their feet, like horses, from sheer exhaustion; they perish, and are found dead in their cages, like so many neglected wild beasts in captivity. But the cangue is a favorite punishment among the judges.—*All the Year Round.*

